# Nigrostriatal pathology with reduced astrocytes in LRRK2 S910/S935 phosphorylation deficient knockin mice

**DOI:** 10.1016/j.nbd.2018.09.003

**Published:** 2018-12

**Authors:** Ye Zhao, Shikara Keshiya, Farzaneh Atashrazm, Jianqun Gao, Lars M. Ittner, Dario R. Alessi, Glenda M. Halliday, Yuhong Fu, Nicolas Dzamko

**Affiliations:** a Central Clinical School, Faculty of Medicine and Health, University of Sydney, Camperdown, NSW 2050, Australia; b School of Medical Sciences, Faculty of Medicine, University of New South Wales, Kensington, NSW 2033, Australia; c Neuroscience Research Australia, Randwick, NSW 2031, Australia; d Dementia Research Centre, Faculty of Medicine and Health Sciences, Macquarie University, Sydney, NSW 2109, Australia; e MRC Protein Phosphorylation and Ubiquitylation Unit, School of Life Sciences, University of Dundee, Dundee, UK

**Keywords:** LRRK2, Phosphorylation, α-Synuclein, Knockin mouse, Striatum, Astrocytes, Dopamine, LRRK2, leucine rich repeat kinase 2, TH, tyrosine hydroxylase, DAT, dopamine transporter, VMAT2, vesicular monoamine transporter 2, GFAP, glial fibrillary acid protein, IBA1, ionized calcium binding adaptor molecule 1, PFF, pre-formed fibrils, PBS, phosphate buffered saline, WT, wild type, KI, S910A/S935A double knockin mice, SN, substantia nigra

## Abstract

Leucine-rich repeat kinase 2 (LRRK2) is genetically implicated in both familial and sporadic Parkinson's disease (PD). Moreover, LRRK2 has emerged as a compelling therapeutic target for the treatment of PD. Consequently, there is much interest in understanding LRRK2 and its role in PD pathogenesis. LRRK2 is constitutively phosphorylated on two serines, S910 and S935, that are required for interaction of LRRK2 with members of the 14-3-3 family of scaffolding proteins. Pathogenic LRRK2 missense mutations impair the phosphorylation of LRRK2 at these sites, but whether this contributes to PD pathology is unclear. To better understand how loss of LRRK2 phosphorylation relates to PD pathology, we have studied double knockin mice in which Lrrk2's serine 910 and 935 have both been mutated to alanine and can therefore no longer be phosphorylated. Nigrostriatal PD pathology was assessed in adult mice, aged mice, and mice inoculated with α-synuclein fibrils. Under all paradigms there was evidence of early PD pathology in the striatum of the knockin mice, namely alterations in dopamine regulating proteins and accumulation of α-synuclein. Striatal pathology was accompanied by a significant decrease in the number of astrocytes in the knockin mice. Despite striatal pathology, there was no degeneration of dopamine neurons in the substantia nigra and no evidence of a PD motor phenotype in the knockin mice. Our results suggest that modulation of LRRK2 serine 910 and 935 phosphorylation sites may have implications for dopamine turnover and astrocyte function, but loss of phosphorylation at these residues is not sufficient to induce PD neurodegeneration.

## Highlights

• LRRK2 S910 and S935 phosphorylation deficient mice have a propensity to accumulate α-synuclein.
• LRRK2 S910 and S935 phosphorylation deficient mice have mild PD pathology with reduced astrocytes.
• Complete loss of LRRK2 S910 and S935 phosphorylation was insufficient to induce neurodegeneration.

## 1 Introduction

Genetic and clinical evidence suggest a pathogenic role for leucine-rich repeat kinase 2 (LRRK2) in both sporadic and familial Parkinson's disease (PD) (Healy et al., 2008; Nalls et al., 2014; Paisan-Ruiz et al., 2004; Zimprich et al., 2004). Exactly how LRRK2 function or dysfunction leads to PD pathogenesis however, remains unclear. LRRK2 is a large 280 kDa protein with both kinase and GTPase activities, as well as leucine-rich repeat, armadillo repeat and WD40 repeat protein-protein interaction domains. Among the first LRRK2 interacting proteins identified were members of the 14-3-3 family of regulatory proteins (Li et al., 2011; Nichols et al., 2010). Six out of the seven 14-3-3 isoforms interact with LRRK2 in a manner dependent on the phosphorylation of LRRK2 at serine (S) 910 and S935, two residues found just prior to the proteins leucine-rich repeat domain (Nichols et al., 2010; Stevers et al., 2017). In overexpressing cell culture models, ablation of S910 and S935 phosphorylation via mutation of these residues to alanine, results in the loss of 14-3-3 binding and the accumulation of LRRK2 in the cytoplasm (Nichols et al., 2010). This suggests a role for S910 and S935 phosphorylation in the regulation of LRRK2's subcellular localization.

There are at least six pathogenic missense mutations in LRRK2 (R1441C, R1441G, R1441H, Y1699C, G2019S and I2020T), that all occur in domains important for regulating LRRK2 catalytic activities (Cookson, 2010). Strikingly, all of these mutations (except for G2019S) result in a loss of LRRK2 S910 and S935 phosphorylation in both cell culture models and knockin mice (Nichols et al., 2010). Overexpressing these loss of phosphorylation pathogenic LRRK2 variants in cell culture results in the accumulation of LRRK2 in cytoplasmic pools (Nichols et al., 2010). More recently, reduced LRRK2 S910 and S935 phosphorylation has also been observed in post mortem brain tissue from patients with idiopathic Parkinson's disease (Dzamko et al., 2017). In this study, a significant reduction in both S910 and S935 phosphorylation was observed in pathologically affected brain regions. That loss of LRRK2 phosphorylation at S910 and S935 occurs in familial and sporadic PD may suggest a potential pathogenic role for these residues in PD.

Somewhat counterintuitively therefore, is the prominent role for the S910 and S935 phosphorylation sites as pharmacodynamic biomarkers for LRRK2 kinase inhibitors. In this regard, it has been established in cells, mice, primates and humans, that LRRK2 kinase inhibitor treatment results in a dose-dependent decrease in LRRK2 S910 and S935 phosphorylation (Deng et al., 2011; Dzamko et al., 2010; Fuji et al., 2015; Perera et al., 2016). The S910 and S935 residues are not direct LRRK2 autophosphorylation sites, but their phosphorylation is indirectly regulated in a LRRK2 kinase activity-dependent manner, potentially via other upstream kinases such as inhibitor of kappa B kinase (IKK) and casein kinase (CK) family members (Chia et al., 2014; Dzamko et al., 2012) as well as upstream phosphatases (Lobbestael et al., 2013). Similar to the above described effects following mutation of S910 and S935 to alanine, LRRK2 inhibitor treatment of overexpressing cell systems also results in the accumulation of LRRK2 in the cytoplasm (Deng et al., 2011; Nichols et al., 2010), as well as increased ubiquitination of LRRK2 leading to its proteosomal degradation (Lobbestael et al., 2016; Zhao et al., 2015). However, accumulating evidence from pre-clinical cell and animal studies suggest that LRRK2 kinase inhibitors may actually have protective effects in some PD models (Atashrazm and Dzamko, 2016; West, 2015). Furthermore, it is established that the six confirmed LRRK2 pathogenic mutations all increase the kinase activity of LRRK2 (Sheng et al., 2012; Steger et al., 2016), and thus there is substantial interest in developing LRRK2 inhibitors as potential PD therapeutics (Chan and Tan, 2017; West, 2017). Thus, both pathological mutations and potential therapeutics alter LRRK2 S910 and S935 phosphorylation, suggesting a better understanding of the physiological roles of these phosphorylation sites is warranted.

To gain further insight into LRRK2 phosphorylation we have studied adult, aged and α-synuclein fibril-inoculated Lrrk2 S910A/S935A double knockin mice, to determine how the loss of Lrrk2 phosphorylation impacts on PD-like nigrostriatal pathology under these different conditions.

## 2 Material and methods

### 2.1 Mice

Lrrk2 S910A/S935A double knockin mice backcrossed onto a C57BL6 background were provided by Dario Alessi, University of Dundee. The mice were re-derived into our animal facility and heterozygous breeding established to provide wild type and double S910A/S935A knockin (KI) littermate mice for study. All animal work was approved by the University of NSW animal ethics committee (approval numbers 15-28B and 16-24A) and experiments performed in accordance with the Australian code for the care and use of animals for scientific purposes. Both male and female mice were used. Mice were maintained on a 12 h light-dark cycle with food and water ad libitum. Different cohorts of mice were studied at 3 months old, 18 months old and following α-synuclein inoculation as outlined in Supplementary Fig. 1. Mice were maintained on a 12 h light-dark cycle with food and water ad libitum. The 18-month-old mice were raised and perfused at the University of Dundee with fixed brain tissue shipped for analysis.

### 2.2 Genotyping

Genomic DNA was purified from mouse tail tissue using digestion buffer (0.05 mM EDTA; pH 8.0, 200 mM NaCl, 100 mM Tris, 0.2% (w/v) SDS) and 0.1 mg/ml of proteinase K at 55 °C overnight. DNA was then precipitated in 100% ethanol and washed with 70% ethanol. Genomic DNA was genotyped using forward (GTGCTTGAAGTTTGATCATAATGC) and reverse (GCATATAGCATGTAGTGTCATCTCC) primers (Sigma) and GoTaq® Master Mix (Promega). SYBR Safe DNA Gel Stain (Invitrogen) was used for PCR product visualization using a Chemidoc MP Imaging system (Biorad). LRRK2 wild-type was identified by a single band at 326  bp, and the homozygote LRRK2 knock-in genotype by a single band at 401 bp (Supplementary Fig. 2).

### 2.3 α-synuclein fibril preparation

Recombinant human monomeric α-synuclein for generating pre-formed fibrils was obtained from Proteos. For generating fibrils for inoculation we followed the supplied protocol, which has recently been published (Polinski et al., 2018). Briefly, upon thawing, the monomeric α-synuclein protein was diluted to 5 mg/ml in sterile Dulbecco's phosphate buffered saline (DPBS) and continuously shaken at 1000 rpm on an orbital shaker placed in a 37 °C incubator for 7 days. After this time, the presence of fibrils was confirmed by Thioflavin T assay. For this assay, Thioflavin T (Sigma) was diluted to 25 μM in DPBS. 95 μl of diluted Thioflavin T was then mixed with 2.5 μl of fibrillar, or the original stock monomeric α-synuclein as a control, in a black 96 well microplate (Greiner) and incubated at room temperature for 5 min. After incubation, the plate was read with excitation and emission settings of 450 nm and 480 nm respectively, using a Polarstar plate reader (BMG Labteck). 25 μl aliquots of fibrillar α-synuclein were then stored at −80 °C and the same preparation was used for all inoculations. Daily before surgery, the fibrillar α-synuclein was diluted to 1 mg/ml in DBPS and sonicated (Branson Sonifer 250 with microtip, output control = 1, Duty cycle = 10%, Time = 30 s) to generate smaller α-synuclein pre-formed fibrils (PFF) for injection.

### 2.4 Transmission electron microscopy

For transmission electron microscopy, 3 μl of 0.1 mg/ml sonicated or un-sonicated PFF samples were applied to glow-discharged 200 mesh, carbon-only, copper grids (Electron Microscopy Sciences) and negatively stained with 2% uranyl acetate (Polysciences). The grids were imaged in a Philips CM120 electron microscope operated at 200 kV with nominal magnification at 66,000× and a defocus range of −1.0 μm to −1.27 μm. Images were collected on a Gatan Ultrascan 4000 CCD camera.

### 2.5 Stereotactic injection

Male and female mice at 3 months of age were anesthetized by intraperitoneal injection of ketamine hydrochloride (75–100 mg/kg of body weight) and xylazine (10–20 mg/kg body weight), and inoculated with 2 μl of PFF (1 mg/ml) or sterile phosphate buffered saline (PBS) by stereotactic injection. Material was injected into the right hemisphere, targeting the dorsal striatum (located +0.2 mm from the Bregma, +2.25 mm from midline and a depth of +2.6 mm from the dura) and injected by a Hamilton syringe at a rate of 0.1 μl per min. The needle was left in situ for at least 10 min to allow injected material to spread into the surrounding parenchymal area. Once fully recovered from anaesthesia, the mice were monitored at least once daily for any sign of post-operative complications for 7 days.

### 2.6 Behavioural testing

To assess the onset and progression of Parkinson's-disease-related symptoms, behavioural tests were performed at baseline, after habituation at the animal facility for 1 week, followed by assessment at 3 and 6 months post stereotactic injection, when the mice were 6 and 9 months old respectively. Tests were performed between 8:00–12:00 in the light-on cycle for two consecutive days, with 30 min break in between each test. Mice were acclimatized to the testing rooms in their home cage for 1 h prior to tests. All testing apparatus and equipment were cleaned with 70% ethanol in between each mouse. Equal numbers of male and female mice were used. General activity, locomotion and anxiety were assessed by open-field (Brooks and Dunnett, 2009). The open-field apparatus (MED Associates Inc.) was an evenly illuminated square arena (43.2 cm × 43.2 cm) with transparent Plexiglas walls and white floor. Mice were individually placed in the corner of the arena. The horizontal activity of each mouse was measured by infra-red beams for 60 min, the first 30 min of which is for habituation activity and the following 30 min for evaluation of locomotor activity. Motor coordination and balance were assessed by rota-rod testing (Brooks and Dunnett, 2009). The mice were placed on the rota-rod apparatus (Columbus Instruments) at an initial speed of 4 rpm. The mice were exposed to 4 trials. Over 300 s the speed gradually increased from 4 to 40 rpm for the first 2 trials and then 4 to 50 rpm for the subsequent 2 trials, with all trials being performed at 10 min intervals. Within this time, the latency of the mice to fall off the rota-rod was recorded. At baseline, the mice were given a training session (4 trials of 4–40 rpm) for acclimatization to the apparatus prior to testing. The mean latency to fall for each speed was used for analysis.

### 2.7 Tissue preparation

For generating tissues for immunoblotting, mice were anesthetized with isoflurane followed by cervical dislocation. Tissues were rapidly excised and snap-frozen in liquid nitrogen for later analysis. For generating tissues for fractionation, 200 mg of fresh mouse tissue was homogenized using a polytron handheld homogenizer. Cytoplasmic, membrane, nuclear, chromatin bound and cytoskeletal fractions were then sequentially extracted using a detergent-based subcellular protein fraction kit (#87790 ThermoFisher) exactly as per manufacturers' instructions. Fractions were snap frozen and stored at −80 °C until analysis by immunoblot. For generating tissues for immunohistochemistry, mice were administered a lethal intraperitoneal dose of sodium pentobarbital (80–100 mg/kg body weight). Transcardial perfusion was then performed with PBS, followed by cold 4% paraformaldehyde (PFA) fixative diluted in PBS (pH 7.4). The brain was removed and placed in 4% PFA for post-fixation overnight at 4 °C. Tail tips were collected from all mice following perfusion and genotypes reconfirmed.

### 2.8 Immunoblotting

Snap-frozen tissues from mouse brain, lung, kidney and spleen were homogenized in buffer consisting of 50 mM Tris.HCL pH 7.5, 1 mM EGTA, 1 mM EDTA, 1 mM sodium orthovanadate, 50 mM sodium fluoride, 5 mM sodium pyrophosphate, 0.27 M sucrose, 1 mM benzamidine, 1 mM PMSF and 1% Triton X-100. Protein concentration was measured using a bicinchoninic acid assay (Pierce BCA Protein Assay Kit, ThermoFisher) and made up to 2 mg/ml in 1 x lithium dodecyl sulfate sample buffer (ThermoFisher) containing 20 μl/ml of 2-mercaptoethanol. Protein lysates were separated by reducing SDS-PAGE. After wet transfer to nitrocellulose membranes, non-specific binding was blocked with 5% skim milk dissolved in TBS buffer containing 0.1% Tween-20 (Sigma) and membranes were then incubated in primary antibodies overnight at 4 °C. After incubation with either horseradish peroxidase (HRP)-conjugated secondary antibodies or Alexa Fluor (AF)-conjugated secondary antibodies, protein detection was visualized using enhanced chemiluminescence reagent (GE Healthcare) and a Chemidoc MP Imaging system (Biorad). Details of antibodies for western blot are provided in supplementary table 1. The intensity level of protein of interest was quantified using ImageLab software (Biorad) and expressed as arbitrary units standardized to β-actin protein levels. Protein levels were then expressed as a percentage relative to the control group, which was set at 100%. Immunoblots are shown in the relevant figures. Students *t*-test was used to compare between the two groups.

### 2.9 Immunohistochemistry

Following post-fixation, tissues were cryo-protected in 5 ml 30% sucrose diluted in PBS at 4 °C for approximately 24 h (time to sink to the bottom of the container). Each cryo-protected brain was then frozen onto a chuck for serial coronal sectioning at 40 μm using a Leica 1850 cryostat (Leica Microsystems). Sections containing the amygdala and all basal ganglia structures were collected from Bregma 1.18 mm to Bregma −4.04 mm with every 1/6 section collected into a series of separate containers of PBS to create 6 series of sections. All midbrain sections were collected and every 1/6 section put into separate containers of PBS to create 6 series of midbrain sections. The Ser129 phosphorylated α-synuclein antibody (clone 81A) was a kind gift from Virginia Lee, University of Pennsylvania and was used as previously described (Luk et al., 2012). Details of the other antibodies used for immunofluorescence labelling are given in supplementary table 2. Immunostaining was carried out simultaneously for each marker in the same region from the same group. Sections were initially incubated in 10 mM sodium citrate buffer (pH 6.0) for 1 h at 85 °C for antigen retrieval. All sections were then washed in PBS and non-specific sites blocked by serum incubation (either 10% goat or 10% donkey serum (both Sigma) depending on secondary antibody used) in 0.3% Triton-X 100 in PBS for 1 h at room temperature. Incubation with primary antibodies was for 2 days at 4 °C followed by PBS washes. Alexa Fluor-conjugated secondary antibodies were used for 2 h at room temperature. Sections were washed, counterstained with DAPI, mounted and coverslipped with anti-fade mounting medium (Dako). Specificity of the immunofluorescence reaction was validated by omitting the primary antibody and observing no fluorescence staining.

### 2.10 Histochemical analyses

Mouse brain structures were defined according to the Mouse Brain in Stereotaxic Coordinates (Paxinos and Franklin, 2012). Regions of interest identified for analyses were the TH or DAT immunoreactive substantia nigra pars compacta (SNpc) assessed in a 1/6 series of 40 μm thick coronal sections (5–7 sections per mouse), the dorsolateral striatum assessed in a 1/6 series of 40 μm thick coronal sections (5–6 sections per mouse) and the cortical nucleus within the corticomedial complex of the amygdala in a 1/6 series of 40 μm thick coronal sections (5–6 sections per mouse). Informative confocal images for each immunofluorescence marker from each region from each group of mice were captured using the same confocal microscope - either a Nikon C2 Basic or Eclipse 90i with NIS-Elements advanced research imaging software or a Zeiss 710 LSM confocal microscope equipped with Zen 2010 software. For each region, the same camera settings were used, including laser intensity, detector sensitivity, averaging times, pixel dwell, etc. For three-dimensional image stacks, the NIS-Elements software automatically set the focal distance. All informative images presented in the figures were processed by Adobe Photoshop (Adobe Systems Inc.) under the same condition (brightness and contrast setting). Prism (Graphpad software) was used for statistical analyses of the number of identified cells with and without α-synuclein immunopositive inclusions, and the intensity of fluorescence labelling, as described below. A p value of 0.05 was considered significant. Student's *t*-test was used to compare between two groups. Two-way ANOVA with Tukey's multiple comparison test was used to compare between the WT and KI mice that had been inoculated with PBS or PFFs.

#### 2.10.1 Quantitation of the number of TH-immunoreactive neurons in the SNpc

All SNpc containing sections from the 3-month-old and the PBS or PFF inoculated mice were analysed stereologically using an Axio Imager M2 fluorescent microscope (Zeiss) and Stereo Investigator 10.0 software (MBF Bioscience). The SNpc region of interest was outlined for sampling, and live focus and an optical fractionator stereological probe (box size = 30 × 30 × 30 μm^3^ sampling 2 cells on average per box; grid sampling array 137 μm × 101 μm with 12.8 ± 0.5 boxes sampled) used to obtain an unbiased stereological sample within this region. Only those neurons whose nuclei fell entirely inside the probes frame or touching the green inclusion borders of the probe were counted. Repeated measures in 25% of sections by the same assessor varied by <6%. For the analysis of brain tissue from 18-month-old mice, a stereological fractionator technique was used with images of the entire SNpc in spaced sections captured at 10× magnification and the number of all TH immunofluorescent neurons determined within each section using the manual count tool in Adobe Photoshop. For each aged mouse, the total number of TH immunoreactive neurons was estimated by multiplying the sum of the number counted per section ×6. Repeated measures in 25% of sections by the same assessor varied by <8%. For all studies, TH neuron counts in KI mice were expressed as a percent of wild type mice, which was set at 100%.

#### 2.10.2 Assessment of the cellular intensity of dopaminergic markers

For the SNpc, DAPI labelled (29 ± 1 average number per mouse) or TH immunoreactive (28 ± 1 average number per mouse) neurons were identified in 40× confocal images, and the DAT and Vmat2 levels determined within each neuron following background correction to the cerebral peduncle using Image J software. Repeated measures in 20% of sections by the same assessor varied by <4%. For the 3-month-old and the PBS or PFF inoculated WT and KI mice, 10 randomly selected non-overlapping 15 × 15 μm^2^ fields from the dorsolateral striatum at 20× or ~0.17 μm/pixel magnification was used for TH, DAT and Vmat2 immunofluorescence. The relative intensity of immunofluorescent synapses was determined following background correction to the corpus callosum using Image J software. Repeated measures in 20% of sections by the same assessor varied by <6%. For 18-month-old KI mice, low magnification images for TH sampling the whole dorsolateral striatum (2.5× or ~4.5 μm/pixel magnification on an Axio Imager M2 fluorescent microscope with 160 × 160 μm^2^ area sampled per mouse), and higher magnification confocal images for DAT (40× or ~0.3 μm/pixel) and Vmat2 (100× or ~0.12 μm/pixel) in 10 randomly selected non-overlapping 15 × 15 μm^2^ fields from the dorsolateral striatum were assessed. The intensity levels of these markers were expressed as the percentage of the average of the WT group, which was set at 100%.

#### 2.10.3 Assessment of α-synuclein immunopositive cytoplasmic and synaptic staining

In the SNpc, α-synuclein was co-stained with TH to examine the proportion of α-synuclein positive cells co-localizing with TH-immunoreactive neurons (21 ± 1 average number sampled per mouse) using Image J software. First, cytoplasmic α-synuclein intensity was identified as that being higher than the average intensity of the sectional background (n = 10 background loci/section) corrected to the background levels in the cerebral peduncle in 40× or ~0.15 μm/pixel magnified confocal images. The Image J JACoP plugin was used to determine the proportion of co-localization. Repeated measures in 20% of sections by the same assessor varied by <4%. For the intensity of the synaptic α-synuclein terminals in the dorsolateral striatum and amygdala, the measured intensity levels were expressed as the percentage of the average of the WT group, which was set at 100%. For the 3-month-old and the PBS or PFF inoculated WT and KI mice, 10 randomly selected non-overlapping 15 × 15 μm^2^ fields from the dorsolateral striatum at 20× or ~0.17 μm/pixel magnification was used for α-synuclein immunofluorescence. The relative intensity of α-synuclein synaptic immunostaining was determined following background correction to the corpus callosum using Image J software. Repeated measures in 20% of sections by the same assessor varied by <5%. In the amygdala, the intensity was assessed in 10 randomly selected non-overlapping 10 × 10 μm^2^ fields in 40× or ~0.3 μm/pixel magnified confocal images. Repeated measures in 15% of sections by the same assessor varied by <5%. For the dorsolateral striatum in the 18-month-old mice, low magnification images for α-synuclein sampling the whole dorsolateral striatum (2.5× or ~4.5 μm/pixel magnification on an Axio Imager M2 fluorescent microscope with 160 × 160 μm^2^ area sampled per mouse) were assessed.

#### 2.10.4 Quantitation of the number of glial cells

DAPI labelled Iba1 immunoreactive microglia were assessed in the SN by sampling two-four captured images/SN section at 40× or ~0.15 μm/pixel magnification (6 ± 1 average microglia per sampled image, 18 ± 3 images per mouse SN). The sampled SN image contained both SNpc and SNpr, that were differentiated for counting microglia by co-labelling with. Microglia were quantified in the dorsolateral striatum by sampling the entire region at 20× or ~0.17 μm/pixel magnification (9 ± 1 average microglia per sampled image, 1 image/section, 5–6 images per mouse dorsolateral striatum). Repeated measures in 20% of sections by the same assessor varied by <4%. DAPI labelled Gfap immunoreactive astrocytes were assessed in the dorsolateral striatum by sampling the entire region at 20× or ~0.17 μm/pixel magnification (4 ± 1 average astrocytes per sampled image, 1 image/section, 5–6 images per mouse dorsolateral striatum), and in the amygdala by sampling the entire cortical nucleus at 40× or ~0.15 μm/pixel magnification (4 ± 1 average astrocytes per sampled image, 1 image/section, 5–6 images per mouse cortical nucleus in the amygdala). Repeated measures in 15% of sections by the same assessor varied by <3%. Glia adjacent to or within the corpus callosum and blood vessels were excluded. The number of immunofluorescent cells was determined within standardized sampled regions using the manual count tool in Image J software and the average number calculated per standardized image view. The number of glia cells was expressed as the percentage of the average of the WT group, which was set at 100%.

## 3 Results

### 3.1 Effect of S910A/S935A KI on Lrrk2 protein, activity and subcellular localization

**Fig. 1:**
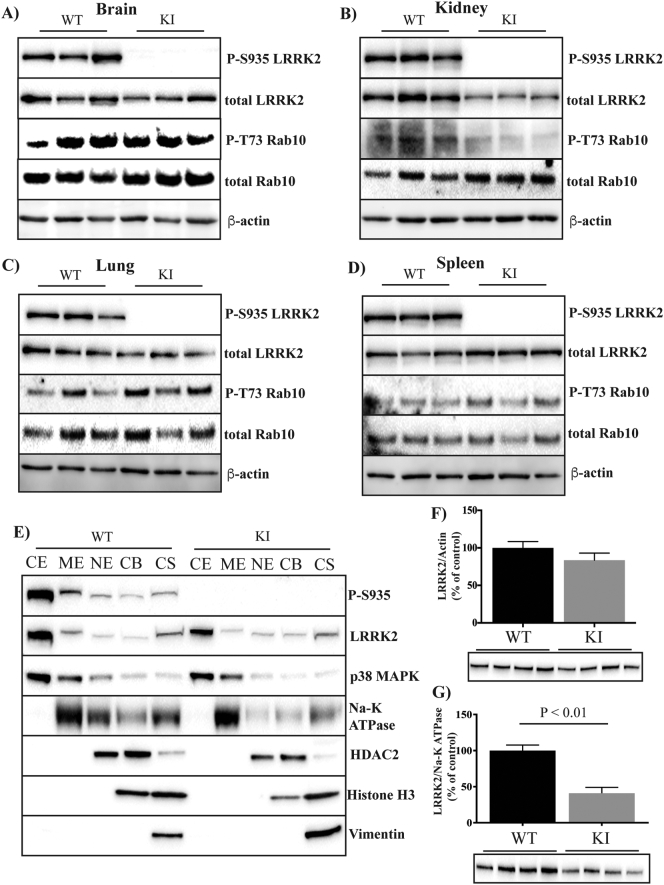
Effect of LRRK2 S910A/S935A mutation on LRRK2 protein, activity and subcellular localization. Lrrk2 phosphorylation at serine 935, total Lrrk2, phosphorylated T73 Rab10 and total Rab10 were assessed by immunoblot in the brain (A), kidney (B), lung (C) and spleen (D) from the adult wild type and Lrrk2 S910A/S935A KI mice at 3 months of age. β-actin was used as a loading control. (E) Following fractionation, Lrrk22 levels were measured by immunoblot in the cytoplasmic extract (CE), membrane extract (ME), nuclear extract (NE), chromatin bound (CB) and cytoskeletal (CS) fractions using brain tissue from the adult wild type and KI mice at 3 months of age. Representative immunoblots are shown from n = 4 mice. Brain protein levels of Lrrk2 were expressed in the cytoplasm (F) and membrane (G) fractions as the percent of the wild type group, which was set at 100%. Quantified immunoblot data are presented as mean ± SEM. Student's *t*-test was used to compare the groups. *Indicates p < .01 compared to wild type.

We first collected tissues from 3-month-old untreated mice to assess the effect that mutating S910A and S935A would have on the expression of endogenous Lrrk2, as well as phosphorylation of its downstream substrate, Rab10. Correct genotyping of the mice was confirmed by complete loss of the S935 phosphorylation site in the KI mice in all tissues examined (Fig. 1A–D). In brain, lung and spleen tissue, levels of total Lrrk2 and phosphorylation of Rab10 T73 were similar between the wild type and KI mice, however in kidney, both total levels of Lrrk2 and Rab10 T73 phosphorylation were reduced in the KI mice (Fig. 1A–D). As knockout of Lrrk2 in kidney has previously been associated with morphological changes (Tong et al., 2010), we examined kidney appearance and weight in the KI mice, but found no difference compared to wild type (Supplementary. Fig. 3). We next aimed to determine if KI mice had an altered subcellular localization of Lrrk2. Subcellular fractionation experiments using brain tissue showed similar levels of Lrrk2 in the nuclear, chromatin bound and cytoskeletal fractions, whereas Lrrk2 levels appeared lower in the cytoplasmic and membrane bound fractions of the KI mice (Fig. 1E). To better quantify the fractionation results, we re-blotted both the cytoplasmic and the membrane-bound fractions with wild type and KI mice side-by-side. This showed a similar level of Lrrk2 in the cytoplasmic fraction (Fig. 1F), but a significantly reduced level of Lrrk2 in the membrane-bound fraction (Fig. 1G) of the KI mice.

### 3.2 Reduced dopamine regulating proteins with increased α-synuclein in adult KI mice striatum

**Fig. 2:**
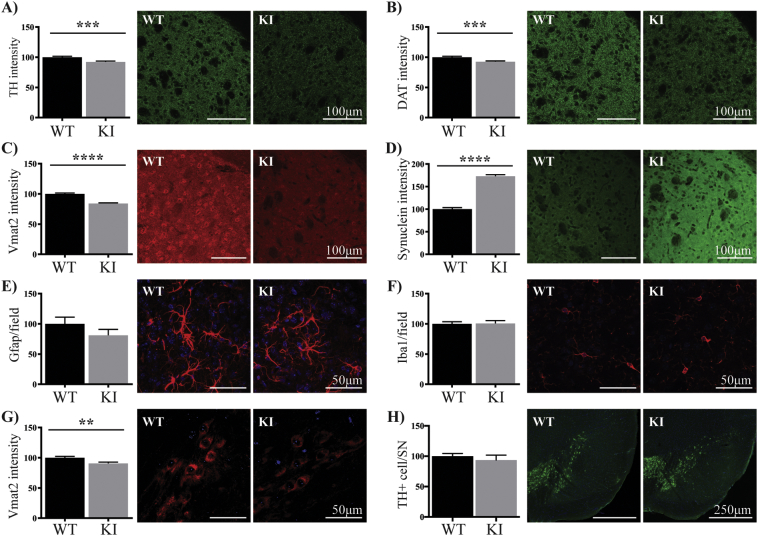
Reduced dopamine regulating proteins with increased α-synuclein in the striatum of adult LRRK2 S910A/S935A KI mice. The intensity of TH (green) (A), DAT (green) (B), Vmat2 (red) (C) and α-synuclein (green) (D) was measured in the dorsolateral striatum of adult WT (n = 8) and KI (n = 8) mice at 3 months of age. (E, F) The number of Gfap (red) (E) and Iba1 (red) (F) positive glia cells were also counted in the dorsolateral striatum. The intensity of Vmat2 (red) (G) and the number of TH (green) positive neurons (H) were measured in the substantia nigra from the same animals. The intensity levels of the immunofluorescent markers and the number of glia cells and TH positive neurons in the substantia nigra were expressed as the per cent of the wild type group, which was set at 100%. The blue staining is DAPI. Data are presented as mean ± SEM. All images are representative of at least n = 6 per mouse. Student's *t*-test was used to compare the groups. ** indicates p < .01, *** indicates p < .001 and **** indicates p < .0001 compared to the wild type mice. (For interpretation of the references to colour in this figure legend, the reader is referred to the web version of this article.)

We next went on to further characterize brain tissue from untreated wild type and KI mice of 3 months of age. Due to its relevance to PD, we focused on α-synuclein and proteins of the nigrostriatal dopamine pathway. In the dorsolateral striatum of the KI mice, we found a significant decrease in staining intensity of the dopamine regulating proteins TH, DAT and Vmat2 (8%, 8% and 16% respectively and all p < .01) as compared to wild type (Fig. 2A–C). This was accompanied by a significant increase (73%, p < .001) in the intensity of α-synuclein staining (Fig. 2D). The number of Gfap positive astrocytes in the striatum of the KI mice trended lower, but were not significantly different (Fig. 2E), whereas the number of Iba1 positive microglia were the same in both genotypes (Fig. 2F). In contrast to the striatum, in the TH positive SNpc neurons, we found only a small decrease in Vmat2 (9%, p < .01, Fig. 2G), whilst the intensity of DAT (Supplementary Fig. 4A) and α-synuclein were unchanged (Supplementary Fig. 4B). Stereological counting revealed no difference between the two groups in the number of TH immunopositive SNpc neurons (Fig. 2H), whilst the number of Iba1 positive microglia in the entire SN were also similar between the two groups (Supplementary Fig. 4C).

### 3.3 Nigrostriatal pathology with reduced astrocytes in aged S910A/S935A KI mice striatum

**Fig. 3:**
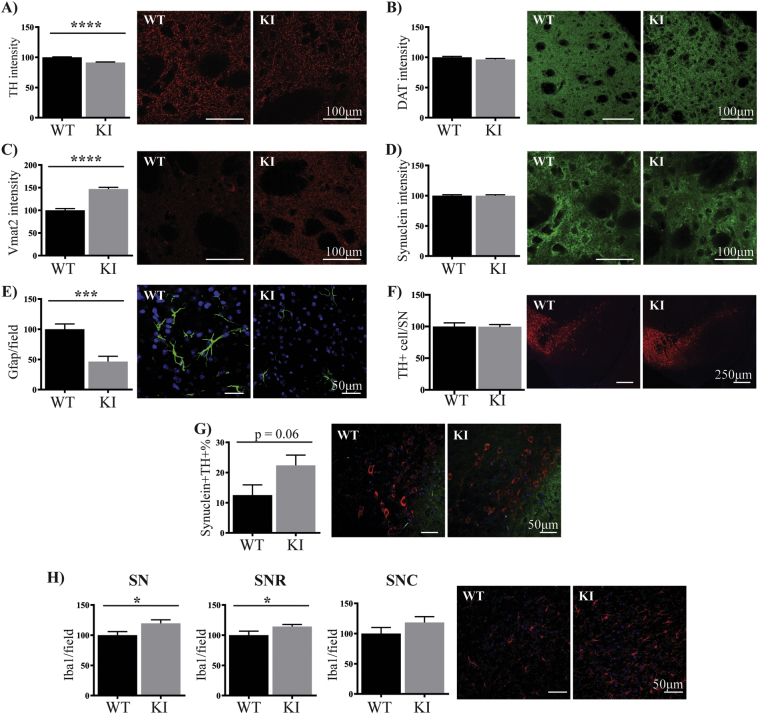
Abnormal dopamine terminals with reduced astrocytes in the striatum of aged LRRK2 S910A/S935A KI mice. The intensity of TH (red) (A), DAT (green) (B), Vmat2 (red) (C) and α-synuclein (green) (D) was measured in the dorsolateral striatum of wild type (n = 8) and KI (n = 8) mice at 18 months of age. The number of Gfap (green) (E) positive astrocytes was also counted in the dorsolateral striatum. The number of TH (red) positive neurons (F) and the proportion of TH (red) positive neurons co-localizing α-synuclein (green) (G) were measured in the substantia nigra from the same animals. The number of Iba1 (red) positive microglia (H) in 40× captured view was measured in the substantia nigra pars reticulata and pars compacta. The intensity levels of the markers and the number of glia cells and TH positive neurons in the substantia nigra were expressed as the per cent of the WT group, which was set at 100%. The blue staining is DAPI. Data are presented as mean ± SEM. All images are representative of at least n = 20 per genotype. Student's *t*-test was used to compare the groups. *** indicates p < .001, and **** indicates p < .0001 compared to the wild type mice. (For interpretation of the references to colour in this figure legend, the reader is referred to the web version of this article.)

To determine whether changes in the nigrostriatal dopamine pathway were altered with age, we next studied brain tissue from wild type and KI mice at an age of 18 months. In the dorsolateral striatum of the aged KI mice, we found a significant decrease in the intensity of TH immunoreactivity (9%, p < .001, Fig. 3A). Intensity levels of DAT were similar between the two genotypes (Fig. 3B), however, Vmat2 immunofluorescence intensity was increased in KI mice (47%, p < .001, Fig. 3C). Although the intensity of α-synuclein in the dorsolateral striatum was similar between wild type and KI groups (Fig. 3D), a small number of abnormal α-synuclein immunopositive inclusions were observed in the KI mice but not in the wild type mice (Supplementary Fig. 5A). These inclusions were located in small, non-TH immunoreactive cells as determined by their association with DAPI-positive nuclei. Intriguingly, there was a significant decrease in the number of Gfap positive astrocytes in the dorsolateral striatum of the KI mice (54%, p < .001, Fig. 3E), whereas the number of Iba1 positive microglia remained unchanged (Supplementary Fig. 5B). In the SNpc, there was no difference either in the number of TH positive neurons (Fig. 3F), or the intensity levels of neuronal DAT (Supplementary Fig. 5C) or Vmat2 (Supplementary Fig. 5D) between the two genotypes. In contrast, there was a non-significant trend for an increased proportion of TH positive SNpc neurons to contain α-synuclein accumulations in the KI mice (of ~50%, p = .06, Fig. 3G). This change was associated with a 20% increase in the number of Iba1 positive microglia in the SN, in particular in the substantia nigra pars reticulata, of the KI group (Fig. 3H).

### 3.4 PFF inoculation and behavioural testing in S910A/S935A KI mice

**Fig. 4:**
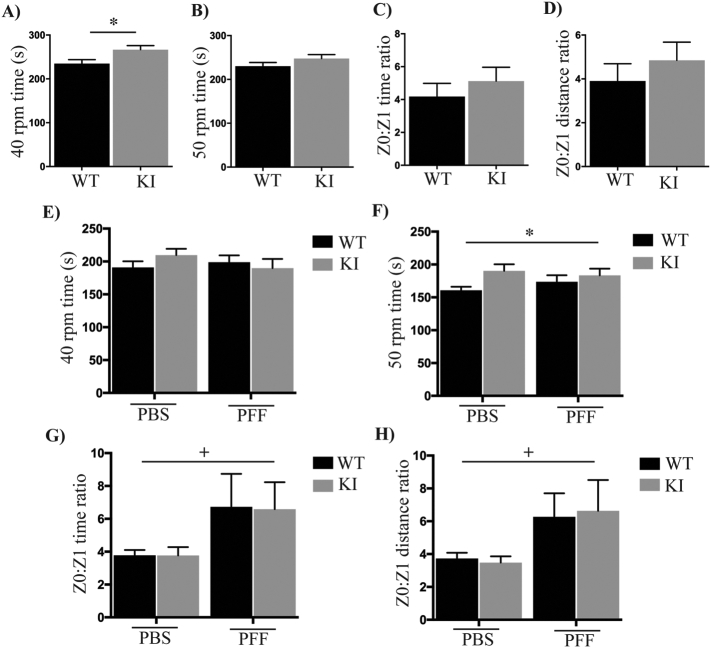
Motor phenotypes in the wild type and KI mice before and after PBS or PFF inoculation. Prior to stereotactic surgery, the mean latency to fall for a maximum speed of 40 rpm (A) and 50 rpm (B) was determined in the rotarod test for both the wild type (n = 43) and KI (n = 39) mice at 3 months of age. Time spent (C) and the total distance travelled in the Zone 0 (edge square) to Zone 1 (centre square) ratio (D) in the open-field test was also assessed. Both the rotarod (E, F) and open field tests (G, H) were again performed at 24 weeks post injection with either phosphate buffered saline (PBS) or α-synuclein fibrils (PFF), when the mice were 9 months of age. Data are presented as mean ± SEM. Data were analysed using Student's *t*-test before surgery, and two-way ANOVA with Tukey's multiple comparisons test after surgery. + indicates p < .05 compared to PBS treatment, * indicates p < .05 compared to the wild type mice.

We next aimed to determine if loss of Lrrk2 S910 and S935 phosphorylation could potentiate α-synuclein pathology induced by intra-striatal injection of PFFs. Prior to surgery, rota-rod and open field tests were conducted using wild type and KI mice at 3 months of age. For the rota-rod test we initially used a maximum speed of 40 rpm, and under these conditions KI mice actually performed significantly better than wild type mice (Fig. 4A). When the rota-rod test was repeated with a maximum speed of 50 rpm, a significant difference was no longer seen between the two genotypes (Fig. 4B). There was also no significant difference between wild type and KI mice in the open field test in terms of time spent in the different zones (Fig. 4C) and the distance covered (Fig. 4D). After establishing the baseline conditions, mice were unilaterally injected into the striatum with α-synuclein PFFs or PBS as a control. PFFs were generated from recombinant monomeric α-synuclein resulting in an ~50-fold increase in thioflavin T fluorescence (Supplementary Fig. 6A). Electron microscopy analysis of fibril morphology indicated sonicated fibrils ranged in size from ~20–300 nm (Supplementary Fig. 6B). Injection into the dorsolateral striatum was also confirmed using 1% methylene blue (Supplementary Fig. 7). Following injection of PBS or PFFs, body weight was recorded weekly. At 12-weeks post injection, when mice were 6 months of age, all mice underwent behavioural testing again. At this time point there was no difference in rota-rod or open field testing and no difference in body weight between the treatment groups or genotypes. By 18 weeks post injection however, PFF injected mice had gained significantly more body weight than PBS injected mice (Supplementary Fig. 8). This was the same for both wild type and KI mice. Behavioural tests were again performed at 24 weeks post injection, when the mice were 9 months old. There were no significant differences between treatment groups or genotypes for the 40 rpm rota-rod test (Fig. 4E). However, for the 50 rpm rota-rod test, KI mice performed significantly better than wild type mice, regardless of whether they received PFF or PBS injections (Fig. 4F). In the open field test, there was a significant difference between the PBS and PFF treatment groups, with PFF injected mice spending more time (Fig. 4G) and covering more distance (Fig. 4H) around the periphery of the open field. This is indicative of an anxiety phenotype in the PFF injected mice, and both wildtype and KI PFF injected mice demonstrated this phenotype to the same extent.

### 3.5 Phosphorylated α-synuclein propagation following pre-formed fibril inoculation

**Fig. 5:**
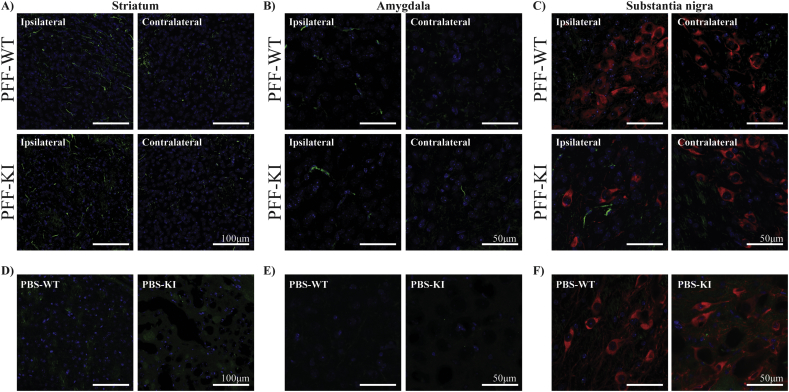
Phosphorylated α-synuclein pathology in the wild type and KI mice before and after PBS or PFF inoculation. Immunofluorescent analysis of Ser129 phosphorylated α-synuclein (green) was performed in both the ipsilateral and contralateral sites of the dorsolateral striatum (A), cortical nucleus of the amygdala (B) and substantia nigra pars compacta (C) in the α-synuclein fibrils (PFF) treated wild type and KI groups at 24 weeks post inoculation. Ser129 phosphorylated α-synuclein was also assessed in the ipsilateral sites of the dorsolateral striatum (D), cortical nucleus of the amygdala (E) and substantia nigra pars compacta (F) in the phosphate buffered saline (PBS) treated group at 24 weeks post inoculation. In the substantia nigra (C,F) the red staining is TH. The blue staining is DAPI. All images are representative of at least n = 3 per mouse and at least n = 4 mice were studied per group. (For interpretation of the references to colour in this figure legend, the reader is referred to the web version of this article.)

To confirm that our exogenous human α-synuclein PFFs could induce pathology of endogenously expressed mouse α-synuclein, immunofluorescent analysis of Ser129 phosphorylated α-synuclein was performed. Fibrillar phosphorylated α-synuclein Lewy neurite-like accumulations were observed in both the ipsilateral (Supplementary Fig. 9A) and contralateral striatum in both the PFF treated wild type and KI mice (Fig. 5A). To determine if α-synuclein pathology had propagated from the striatum we also assessed Ser129 phosphorylated α-synuclein in the amygdala and SN. In the amygdala, the same fibrillar phosphorylated α-synuclein accumulations were present in the ipsilateral side of both the wild type and KI PFF treated groups (Fig. 5B). However, in the contralateral amygdala, fibrillar accumulations were only observed in the PFF injected KI mice, and not in the wild type mice (Fig. 5B). Again, in the ipsilateral SN, α-synuclein accumulations were observed in the PFF injected KI mouse only (Fig. 5C). In both the amygdala (Supplementary Fig. 9B) and SN (Supplementary Fig. 9C), these accumulations were always near DAPI-positive nuclei. Lewy neurite and Lewy body-like pathologies were absent in ipsilateral striatum (Fig. 5D), amygdala (Fig. 5E) and SN (Fig. 5F) of the PBS injected wild type and KI mice. These results show that the PFF inoculations induced α-synuclein pathology and suggest that the propagation of α-synuclein pathology is potentiated in the KI mice.

### 3.6 Quantification of total α-synuclein following pre-formed fibril inoculation

**Fig. 6:**
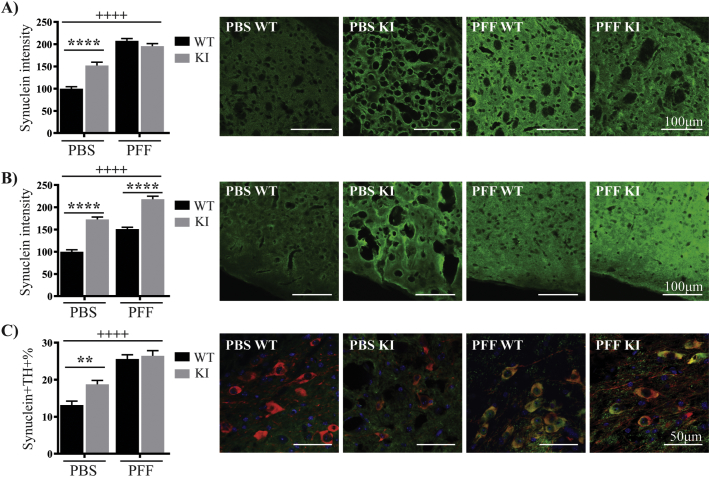
Increased total α-synuclein intensity following PFF inoculation. Immunofluorescent analysis of total α-synuclein (green) was performed in the ipsilateral dorsolateral striatum (A) and the cortical nucleus of the amygdala (B) for both the phosphate buffered saline (PBS) and α-synuclein fibrils (PFF) treated wild type and KI mice at 24 weeks post inoculation. The intensity values were expressed as the percent of the PBS treated wild type group, which was set at 100%. (C) The proportion of TH positive neurons (red) co-localizing α-synuclein (green) in the ipsilateral substantia nigra pars compacta was also assessed for each group. The blue staining is DAPI. Data are presented as mean ± SEM. All images are representative of at least n = 3 per mouse, with at least 8 mice studied per group. Two-way ANOVA with Tukey's multiple comparisons test was used to compare the groups. ++++ indicates p < .0001 compared to PBS treatment and ** indicates p < .01 and **** indicates p < .0001 compared to the wild type mice. (For interpretation of the references to colour in this figure legend, the reader is referred to the web version of this article.)

We next measured the intensity of total α-synuclein staining in the ipsilateral striatum (Fig. 6A), amygdala (Fig. 6B), and in SNpc TH positive neurons (Fig. 6C) for both the PFF and PBS inoculated wild type and KI mice. In all three brain regions investigated, the α-synuclein intensity was higher in the KI mice injected with PBS, compared to the wild type mice injected with PBS (Fig. 6A–C). Again, in all three brain regions, the α-synuclein intensity was higher in the PFF injected mice compared to the PBS group (Fig. 6A–C). In comparing the PFF inoculated groups, the α-synuclein intensity was significantly elevated in the cortical nucleus of the amygdala of the PFF injected KI mice compared to the PFF injected wild type mice, but there was no difference between the two groups in α-synuclein intensity in the striatum or SN (Fig. 6A–C). In agreement with our phosphorylated α-synuclein results, inclusions were again observed in the striatum (Supplementary Fig. 9D) and SN (Supplementary Fig. 9E) of the PFF injected KI mice, but not wild type mice.

### 3.7 Nigrostriatal pathology with reduced astrocytes in PFF inoculated S910A/S935 KI mice striatum

**Fig. 7:**
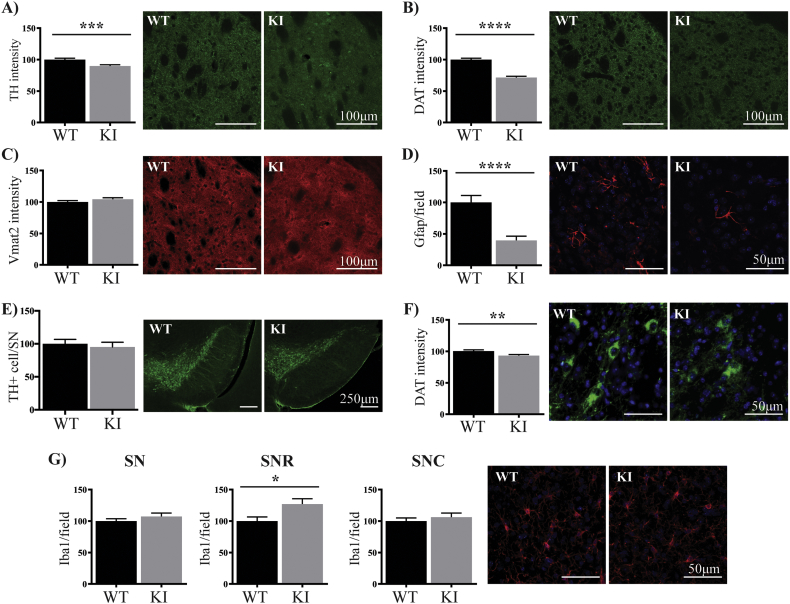
Changes in nigrostriatal pathology with reduced striatal astrocytes in PFF inoculated KI mice. The intensity of TH (green) (A), DAT (green) (B) and Vmat2 (red) (C) was measured in the dorsolateral striatum of the α-synuclein fibrils (PFF) treated wild type and KI mice at 9 months of age. The number of Gfap (red) (D) positive astrocytes were also counted in the dorsolateral striatum. The number of TH (green) positive (E) neurons was counted in the substantia nigra of the wild type and KI mice. The intensity of DAT (green) (F) was measured in the substantia nigra neurons. The number of Iba1 (red) positive microglia (G) in 40× captured view was also measured in the substantia nigra pars reticulata and pars compacta. The blue staining is DAPI. All data are presented as mean ± SEM. The intensity levels of the markers and the number of glia cells and TH positive neurons in the substantia nigra were expressed as the per cent of the wild type group, which was set at 100%. All images are representative of at least n = 4 per mouse, with at least 8 mice studied per group. Student's *t*-test was used to compare the groups. ** indicates p < .01, *** indicates p < .001 and **** indicates p < .0001 compared to the wild type mice. (For interpretation of the references to colour in this figure legend, the reader is referred to the web version of this article.)

Having confirmed successful induction of α-synuclein pathology with the PFFs, we then examined their effect on the nigrostriatal dopamine pathway in the wild type and KI mice. In the striatum of the PFF injected KI mice, the intensities of TH immunoreactivity (Fig. 7A), and in particular DAT immunoreactivity (Fig. 7B), were both significantly decreased compared to wild type PFF injected mice (10% and 28% respectively, both p < .01). Vmat2 levels however, were unchanged between the two genotypes (Fig. 7C). Again, there was a significant reduction in the number of Gfap positive astrocytes in the striatum of the KI mice (60%, p < .001, Fig. 7D), whereas the number of Iba1 positive microglia were unchanged (Supplementary Fig. 10A). In PFF injected mice, there was no difference in the number of TH positive SNpc neurons between the two genotypes (Fig. 7E) and no difference in the intensity levels of VMAT2 in SNpc neurons (Supplementary Fig. 10B). There was however, a significant decrease in DAT intensity in SNpc neurons of the KI PFF injected mice compared to wild type PFF injected mice (7%, p < .01, Fig. 7F). In the SN as a whole, there was also no difference in Iba1 positive microglia between the two PFF injected genotypes. However, there was a significant increase in microglia in the KI PFF injected mice if only the substantia nigra pars reticulata was assessed (Fig. 7G). In the cortical nucleus of the amygdala, the number of Gfap positive astrocytes was not different between the two PFF injected genotypes (Supplementary Fig. 10C).

## 4 Discussion

In this study we have utilized KI mice in which the LRRK2 phosphorylation sites, S910 and S935, have been mutated to alanine and can therefore no longer be phosphorylated. Our aim was to determine the impact of loss of LRRK2 phosphorylation on brain pathology relevant to PD. Generation and initial validation showing loss of S910 and S935 phosphorylation in the double knockin (KI) mice has been previously described (Ito et al., 2016). Mouse embryonic fibroblasts (MEFs) derived from the KI mice show similar levels of total Lrrk2 and in vitro kinase activity compared to wild type MEFs, although phosphorylation of the Lrrk2 substrate Rab10 (Steger et al., 2016) was markedly reduced in KI MEFs when assessed by phos-tag assay (Ito et al., 2016). Our characterization of tissues from the KI mice suggest an element of tissue specificity when it comes to effects of loss of Lrrk2 phosphorylation. In particular, we found a decrease in total Lrrk2 in kidney tissue from the KI mice whereas in brain, spleen and lung the levels of Lrrk2 were similar between genotypes. The reduced levels of Lrrk2 in kidney did not cause the morphological kidney phenotype previously seen with the complete loss of Lrrk2 (Tong et al., 2012; Tong et al., 2010), at least in the adult mice studied. We also found no difference in the phosphorylation of Rab10 in tissues outside of kidney, suggesting that Lrrk2 kinase activity remains largely intact in the KI mice. Why this differs from the reduced Rab10 phosphorylation seen in KI MEFs is unknown, but could be due to different cell types/tissues analysed and/or different detection methodologies. Both MEFS and kidney also have relatively high expression levels of LRRK2 which may also contribute to a specific effect in these cell types. In regards to the difference in detection methodology we have employed a new sensitive and specific T73 Rab10 phosphorylation antibody (Lis et al., 2018) that allows phosphorylation only at that particular residue to be measured. We also attempted to assess if loss of Lrrk2 phosphorylation could alter the enzymes subcellular localization. To do this we used a tissue subcellular fractionation protocol, as there are no widely accepted methods to assess Lrrk2 subcellular localization by microscopy (Davies et al., 2013). In most tissues and in most fractions the levels of Lrrk2 were similar in wild-type and KI mice, suggesting no dramatic shift in enzyme localization due to loss of phosphorylation. In brain tissue of KI mice however, there was a significant reduction in Lrrk2 levels in the membrane fraction. Lrrk2 has been associated with intracellular membrane bound organelles including endosomes and lysosomes (Roosen and Cookson, 2016) and increasing Lrrk2 S910/S935 phosphorylation following activation of toll-like receptors has been reported to increase the levels of Lrrk2 in the membrane fraction (Schapansky et al., 2014). Our data support a role for S910/S935 phosphorylation in the regulation of Lrrk2 localization, at least in brain, but studies under conditions known to induce phosphorylation and translocation may better reveal the extent to which phosphorylation is required for this likely dynamic process.

We next investigated nigrostriatal PD pathology in adult (3 months) and aged (18 months) wild type and homozygous KI mice, as well as in both genotypes following striatal inoculation with saline or PFF α-synuclein. In the 3-month old mice we found subtle but significantly reduced fluorescence intensity for the dopamine regulating proteins TH, DAT and Vmat2 in the striatum. In the SNpc at this timepoint, Vmat2 intensity was reduced but DAT was unchanged between genotypes. At 18 months of age however, DAT intensity was now unchanged in the striatum of the KI mice, whilst Vmat2 intensity had increased in the KI mice. At this time point Vmat2 was unchanged in the SNpc. These results suggest an age-dependent compensation in dopamine regulating proteins in order to maintain homeostasis. Consistent with compensated dopamine homeostasis, KI mice showed no behavioural phenotypes and there was no evidence of degeneration of SNpc TH neurons under any conditions. Interestingly we also found reduced DAT intensity in the KI mice in both the striatum and SNpc following α-synuclein PFF inoculation. Both R1441C and R1441G knockin mice, which exhibit increased LRRK2 kinase activity, but with ~50% and ~80% loss of LRRK2 phosphorylation respectively have been studied previously (Giesert et al., 2017; Liu et al., 2014; Tong et al., 2009). Consistent with our results, these mice do not develop neurodegeneration or behavioural deficits, although aged R1441G mice display gait impairments which we did not assess in our model (Liu et al., 2014). All three studies also report no change in striatal TH, Vmat2 and DAT levels. However, R1441C mice have been reported to exhibit impaired dopamine release following amphetamine stimulation (Tong et al., 2009), and R1441G mice have been reported to have impaired dopamine uptake following reserpine treatment (Liu et al., 2014). This suggests an underlying defect in the regulation of proteins involved in dopamine turnover, which can be exacerbated following challenge. Our results from mice with normal brain Lrrk2 kinase activity but 100% loss of Lrrk2 phosphorylation, suggest that loss of S910 and S935 phosphorylation may contribute to these phenotypes.

An intriguing aspect of our study was the propensity for the KI mice to develop α-synuclein pathology. We found increased α-synuclein intensity in the striatum of KI mice at 3 months of age. Increased α-synuclein intensity was also observed in the 9 month old KI mice that had received striatal injections of saline. In the 18 month old KI mice, α-synuclein intensity was not increased, but α-synuclein punctate accumulations could be observed in the striatum. The result was the same for KI mice following α-synuclein PFF inoculation, with the addition that α-synuclein inclusions could also be seen in the SN and amygdala. Moreover α-synuclein pathology could be detected in the contralateral amygdala, only in the KI mice, suggesting a greater degree of propagation from the inoculation site. These findings differ from the lack of α-synuclein pathology observed in R1441C and R1441G knockin mice (Giesert et al., 2017; Liu et al., 2014; Tong et al., 2009), although we note that increased Ser129 phosphorylated α-synuclein, along with altered levels of DAT and Vmat2, has been observed in aged G2019S knockin mice (Longo et al., 2017). One potential explanation for these results is the astrocyte deficits seen in the phosphorylation deficient KI mice. Increasing evidence suggests that astrocytes may play an important role in clearing pathological α-synuclein in PD (Cavaliere et al., 2017; Lindstrom et al., 2017; Loria et al., 2017; Morales et al., 2017; Rostami et al., 2017), and therefore a reduction in astrocytes may promote α-synuclein accumulation and propagation. We found a significant age-dependent reduction in Gfap positive astrocyte numbers in the striatum of the KI mice, which is also the site where α-synuclein PFFs were inoculated. Little is known about LRRK2 function in astrocytes, although post mortem PD brain analysis suggests that LRRK2 is indeed expressed in astrocytes (Dzamko et al., 2017), and overexpressing pathogenic mutation forms of LRRK2 impairs the lysosomal degradation capacity of primary mouse astrocytes (Henry et al., 2015).

Although PFF inoculation induced α-synuclein pathology, the extent of the pathology was insufficient to cause degeneration of substantia nigra neurons in this particular study. Recently, the importance of the biochemical properties of α-synuclein fibrils in mediating neurotoxicity have become better understood, with fibril size, concentration and even mouse strain being important (Abdelmotilib et al., 2017; Tarutani et al., 2016). Electron microscopy analysis of our fibrils indicated a size range of ~20–300 nm, with a large proportion being longer in size. Strain comparison studies suggest that this may be sufficient to cause neurodegeneration in mice on a C3H/HeJ background (Abdelmotilib et al., 2017), the same strain originally used by Luk et al. to demonstrate fibril-mediated neurotoxicity (Luk et al., 2012). However, inclusion formation and degeneration of substantia nigra neurons in C57BL6 mice, the background strain of the currently studied mice, requires a predominantly <50 nm fibril preparation (Abdelmotilib et al., 2017). Thus, our fibril preparations may not have been optimal for assessing α-synuclein mediated neurodegeneration, but even in aged mice we found no evidence of dopaminergic neuron loss suggesting that the complete loss of LRRK2 phosphorylation is not sufficient on its own to promote neurodegeneration.

In conclusion, understanding LRRK2 pathobiology has been complicated by the dynamic interaction between the enzymes dual GTPase and kinase activities that also feedback to regulate LRRK2 phosphorylation/localization and potentially even levels of LRRK2 itself. The current Lrrk2 S910A/S935A KI mice provide an opportunity to better understand the role of these residues in LRRK2 function, without also perturbing the enzymatic activities. Our results suggest a role for these phosphorylation sites in regulating proteins important for dopamine turnover, and for regulating astrocyte numbers, at least in the striatum, with potential implications for α-synuclein pathology. It is important to note however, that the complete loss of Lrrk2 phosphorylation is beyond what is seen with pathogenic mutations and in idiopathic postmortem brain, and likely beyond what can be achieved with LRRK2 kinase inhibitors. Indeed, LRRK2 inhibitors remain an exciting therapeutic prospect for PD and it may be of interest to determine if any phenotypes in the Lrrk2 S910A/S935A knockin mice can be reversed with inhibitor treatment.

The following are the supplementary data related to this article.

Supplementary tables Image 2

Supplementary figures
Supplementary Fig. 1 Schematic illustration of the studies in LRRK2 S910A/S935A double knockin mice. Experiments performed in different cohorts of mice at 3 months old (baseline), 18 months old (aged) and following α-synuclein PFF or PBS inoculation.
Supplementary Fig. 2. Confirmation of mutation status. PCR followed by SYBR green staining was used to identify wild type (single 326 bp band), homozygote Lrrk2 S910A/S935A KI mice (single 401 bp band), and heterozygous mice (both the 326 and 401 bp bands) from genomic tail tip DNA.
Supplementary Fig. 3. Kidney weight and appearance in adult wild type and KI mice. Gross kidney weight (A), and kidney weight as a percentage of body weight (B), were measured in adult wild type (n = 11) and KI (n = 11) mice at 3 months of age. Data are presented as mean ± SEM. Student's *t*-test was used to compare the groups. (C) Representative images of the kidney appearance between the wild type and KI mice.
Supplementary Fig. 4. No difference in DAT, α-synuclein or microglia in the substantia nigra of adult KI mice.
The intensity of DAT (green) (A) and the proportion of TH (red) positive neurons co-localizing α-synuclein (green) (B) were measured in the substantia nigra from wild type and KI mice at 3 months of age. The number of Iba1 (red) positive microglia (C) were also measured in 40× captured views of the substantia nigra pars reticulata and pars compacta, as well as the total number in the substantia nigra. Data are presented as mean ± SEM. The intensity levels of DAT and the number of microglia were expressed as the percent of the wild type group, which was set at 100%. All images are representative of at least n = 12 per mouse, with at least n = 8 mice studied per group. The blue staining is DAPI. Student's *t*-test was used to compare the groups.
Supplementary Fig. 5. Immunopositive α-synuclein inclusions in the striatum of aged KI mice. (A) Representative images of α-synuclein (green) positive inclusions are shown from 100× captured Z stacks generated in the aged KI mice striatum. An orthogonal view (left) and a three-dimensional rotation view (right) displayed that these inclusions were near DAPI-positive nuclei (blue), but not found in TH positive dopamine terminals (red). (B-D) The number of Iba1 (red) (B) positive microglia was counted in the dorsolateral striatum. The intensity of DAT (green) (C) and Vmat2 (red) (D) was measured in substantia nigra neurons of wild type and KI mice at 18 months of age. Data are presented as mean ± SEM. The number of microglia and the intensity levels were expressed as the percent of the wild type group, which was set at 100%. All images are representative of at least n = 20 per genotype with at least n = 8 mice studied per group. The blue staining is DAPI. Student's *t*-test was used to compare the groups. * indicates p < .05 compared to the wild type mice.
Supplementary Fig. 6. Characterization of α-synuclein fibril preparation.
A) A thioflavin T assay was used to determine the increase in fluorescence intensity of α-synuclein following 7 days of shaking compared to the original monomeric protein. B) transmission electron microscopy was used to assess fibril morphology before and after shaking. Images are representative of n = 10 for each condition.
Supplementary Fig. 7. Confirmation of PFF injection into the dorsolateral striatum. Traced injection of PFF with 1% methylene blue was used to examine whether or not there was successful inoculation into the right side of the dorsolateral striatum. Cervical dislocation of the mice was performed post injection, and then the striatum of fresh-frozen brain was sliced in the coronal plane using a Leica 1850 cryostat. Representative images are shown in serial sections from Bregma 1.09 mm to Bregma −0.11 mm.
Supplementary Fig. 8. Body weight gain over 24 weeks following PFF and PBS inoculation. Body weight was measured prior to surgery and every week post α-synuclein injection until tissue collection after 24 weeks. Body weight data was then used to determine the percentage of weight gain over time for both the wild type and KI mice treated with phosphate buffered saline (PBS) or α-synuclein fibrils (PFF). Data are presented as mean ± SEM with n = 20 mice per group. Two-way ANOVA with Tukey's multiple comparisons test was used to compare the groups. * indicates p < .05 compared to PBS treatment.
Supplementary Fig. 9. α-synuclein accumulations pathology in LRRK2 KI mice. (A) Representative images of S129 phosphorylated α-synuclein positive accumulations are shown from 100× captured Z stacks generated from the ipsilateral striatum of the PFF treated wild type and KI mice. (B) Representative images of S129 phosphorylated α-synuclein positive accumulations are shown from 40× captured Z stacks generated from the ipsilateral amygdala of the PFF treated wild type and KI mice. (C) A representative rotation view of S129 phosphorylated α-synuclein accumulations is shown from 40× captured Z stacks generated from the ipsilateral substantia nigra of the PFF treated KI mice. These accumulations were always near DAPI-positive nuclei, but not found in TH (red) positive substantia nigra neurons. For A – C pathology was assessed in n = 4 mice per group. (D) A representative 100× captured Z stack image of an α-synuclein positive inclusion in the ipsilateral striatum of the PFF treated KI mice. (E) A representative rotation view of an α-synuclein positive inclusion is shown from 40× captured Z stacks generated in the ipsilateral substantia nigra of the PFF treated KI mice. These accumulations were always near DAPI-positive nuclei, but not found in TH (red) positive substantia nigra neurons. For D – E pathology was assessed in n = 8 mice blue group. The blue staining in all images is DAPI.
Supplementary Fig. 10. No difference in striatal microglia or amygdala astrocytes in PFF inoculated S910A/S935A KI mice. The number of Iba1 (red) (A) positive microglia was counted in the dorsolateral striatum from wild type and KI mice at 9 months of age. The intensity of Vmat2 (red) (B) was measured in the substantia nigra from the same mice. The number of Gfap (red) positive astrocytes (C) in a 40× captured view was measured in the cortical nucleus of the amygdala. The blue staining is DAPI. The number of glia cells and the intensity levels were expressed as the percent of the wild type group, which was set at 100%. Data are presented as mean ± SEM. All images are representative of at least n = 8 per mouse, with at least n = 8 mice studied per group. Student's *t*-test was used to compare the groups. * indicates p < .05 compared to the WT mice. Image 1
