# Supplementary material for: Nigrostriatal pathology with reduced astrocytes in LRRK2 S910/S935 phosphorylation deficient knockin mice
Source: Neurobiol Dis. 2018 Dec;120:76–87. doi: 10.1016/j.nbd.2018.09.003 (PMC6197399; doi:10.1016/j.nbd.2018.09.003)
Supplement: Supplementary file 1 — Supplementary tables [file mmc1.docx]

| **Antibody** | **Description** | **Species** | **Clone or catalogue#** | **Dilution** |
| --- | --- | --- | --- | --- |
| **LRRK2** | Total LRRK2 | Mouse monoclonal | NeuroMab, #N241A/34 | 1:1000 |
| **pSer-935 LRRK2** | Phosphorylated LRRK2 | Rabbit monoclonal | Abcam, #UDD2 10(12) | 1:1000 |
| **pT73 Rab10** | LRRK2 substrate | Rabbit monoclonal | Abcam, #ab230261 | 1:1000 |
| **Rab10** | LRRK2 substrate | Rabbit monoclonal | Cell Signaling Technology, #D36C4 | 1:1000 |
| **p38 MAPK** | Cytoplasmic protein | Rabbit polyclonal | Cell Signaling Technology, #9212S | 1:1000 |
| **Sodium Potassium ATPase** | Plasma Membrane protein | Rabbit monoclonal | Abcam, #EP1845Y | 1:50,000 |
| **HDAC2** | Nuclear protein | Rabbit polyclonal | Cell Signaling Technology, #2540S | 1:1000 |
| **Histone H3** | Chromatin-bound protein | Rabbit polyclonal | Abcam, #ab1791 | 1:10,000 |
| **Vimentin** | Cytoskeleton protein | Rabbit monoclonal | Abcam, #EPR3776 | 1:1000 |
| **β-Actin** | Cytoplasm loading control | Mouse monoclonal | Abcam, #AC-15 | 1:10,000 |
| **Donkey anti-mouse AF647** | Secondary antibody | Polyclonal | Abcam, #ab150107 | 1:5000 |
| **Goat anti-mouse HRP** | Secondary antibody | Polyclonal | Bio-Rad, #170-6516 | 1:5000 |
| **Goat anti-rabbit HRP** | Secondary antibody | Polyclonal | Bio-Rad, #170-6515 | 1:5000 |

**Supplementary Table 1. Primary and secondary antibodies used for immunoblotting in this study.**

Catalogue and application details for all primary and secondary antibodies used for immunoblotting in this study.

| **Antibody** | **Description** | **Species** | **Clone or catalogue#** | **Dilution** |
| --- | --- | --- | --- | --- |
| **TH** | Dopamine neurons | Mouse monoclonal | Sigma, #TH-16 | 1:1000 |
| **TH** | Dopamine neurons | Rabbit polyclonal | Chemicon, #AB152 | 1:1000 |
| **DAT** | Dopamine transporters | Rat monoclonal | Millipore, #DAT-Nt | 1:1000 |
| **VMAT2** | Vesicular monoamine transporters | Goat polyclonal | Abcam, #ab87594 | 1:1000 |
| **VMAT2** | Vesicular monoamine transporters | Goat monoclonal | Everest, #SLC18A2 | 1:250 |
| **α-Synuclein** | Synapses | Mouse monoclonal | BD biosciences, #42/α | 1:100 |
| **GFAP** | Astrocytes | Mouse monoclonal | Sigma, #G-A-5 | 1:400 |
| **GFAP** | Astrocytes | Rabbit polyclonal | Sigma, #G9269 | 1:100 |
| **IBA1** | Microglia | Goat polyclonal | Abcam, #ab5076 | 1:1000 |
| **Donkey anti-mouse AF488** | Secondary antibody | Polyclonal | Invitrogen,  #A-21202 | 1:400 |
| **Donkey anti-mouse AF594** | Secondary antibody | Polyclonal | Invitrogen,  #A-21203 | 1:400 |
| **Donkey anti-rabbit AF488** | Secondary antibody | Polyclonal | Invitrogen,  #A-21206 | 1:400 |
| **Donkey anti-rabbit AF594** | Secondary antibody | Polyclonal | Invitrogen,  #A-21207 | 1:400 |
| **Donkey anti-goat AF594** | Secondary antibody | Polyclonal | Invitrogen,  #A-11058 | 1:400 |
| **Donkey anti-rat AF488** | Secondary antibody | Polyclonal | Invitrogen,  #A-21208 | 1:400 |
| **Goat anti-mouse AF488** | Secondary antibody | Polyclonal | Invitrogen,  #A-11001 | 1:400 |
| **Goat anti-rabbit AF594** | Secondary antibody | Polyclonal | Invitrogen,  #A-11012 | 1:400 |

**Supplementary Table 2. Primary and secondary antibodies used for immunofluorescence in this study.**

Catalogue and application details for all primary and secondary antibodies used for immunofluorescence staining in this study. Some primary antibodies were from two companies due to the availability or double labelling strategy.
