# Supplementary material for: Nigrostriatal pathology with reduced astrocytes in LRRK2 S910/S935 phosphorylation deficient knockin mice
Source: Neurobiol Dis. 2018 Dec;120:76–87. doi: 10.1016/j.nbd.2018.09.003 (PMC6197399; doi:10.1016/j.nbd.2018.09.003)
Supplement: Supplementary file 2 — Supplementary figures Supplementary Fig. 1 Schematic illustration of the studies in LRRK2 S910A/S935A double knockin mice. Experiments performed in different cohorts of mice at 3 months old (baseline), 18 months old (aged) and following α-synuclein PFF or PBS inoculation. Supplementary Fig. 2. Confirmation of mutation status. PCR followed by SYBR green staining was used to identify wild type (single 326 bp band), homozygote Lrrk2 S910A/S935A KI mice (single 401 bp band), and heterozygous mice (both the 326 and 401 bp bands) from genomic tail tip DNA. Supplementary Fig. 3. Kidney weight and appearance in adult wild type and KI mice. Gross kidney weight (A), and kidney weight as a percentage of body weight (B), were measured in adult wild type (n = 11) and KI (n = 11) mice at 3 months of age. Data are presented as mean ± SEM. Student's t-test was used to compare the groups. (C) Representative images of the kidney appearance between the wild type and KI mice. Supplementary Fig. 4. No difference in DAT, α-synuclein or microglia in the substantia nigra of adult KI mice. The intensity of DAT (green) (A) and the proportion of TH (red) positive neurons co-localizing α-synuclein (green) (B) were measured in the substantia nigra from wild type and KI mice at 3 months of age. The number of Iba1 (red) positive microglia (C) were also measured in 40× captured views of the substantia nigra pars reticulata and pars compacta, as well as the total number in the substantia nigra. Data are presented as mean ± SEM. The intensity levels of DAT and the number of microglia were expressed as the percent of the wild type group, which was set at 100%. All images are representative of at least n = 12 per mouse, with at least n = 8 mice studied per group. The blue staining is DAPI. Student's t-test was used to compare the groups. Supplementary Fig. 5. Immunopositive α-synuclein inclusions in the striatum of aged KI mice. (A) Representative images of α-synuclein (green) positive inclusions are [file mmc2.pdf]

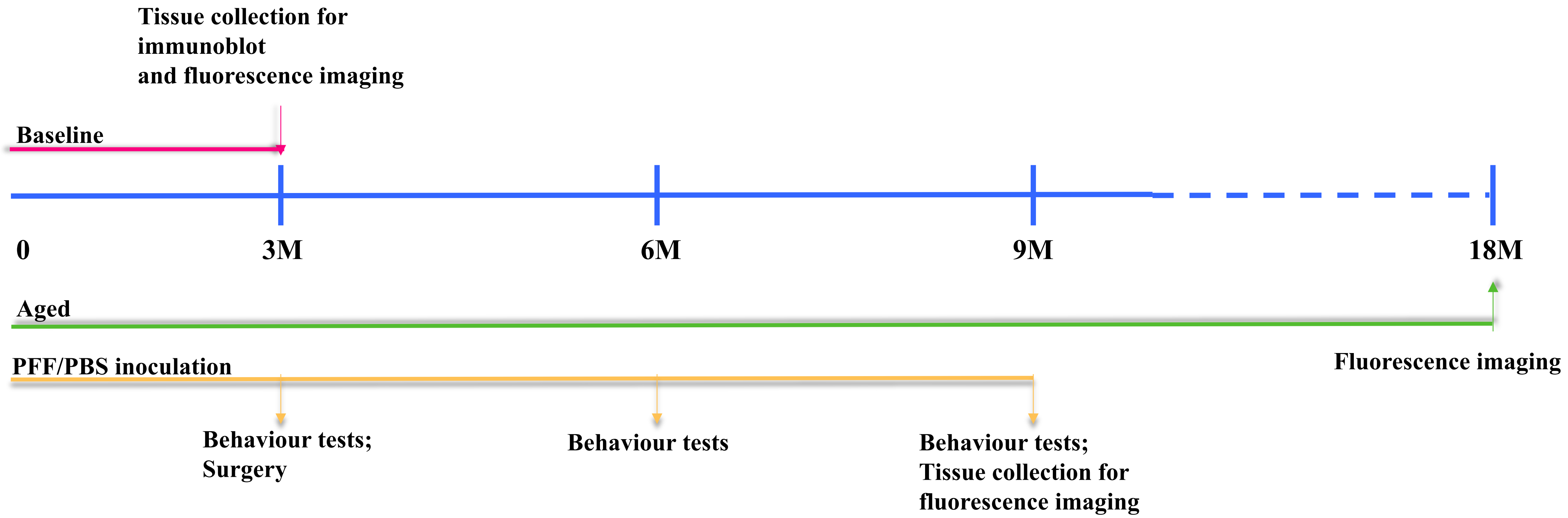
Tissue collection for
immunoblot
and fluorescence imaging
Baseline
0
3M
6M
9M
18M
Aged
PFF/PBS inoculation
Fluorescence imaging
Behaviour tests;
Surgery
Behaviour tests
Behaviour tests;
Tissue collection for
fluorescence imaging

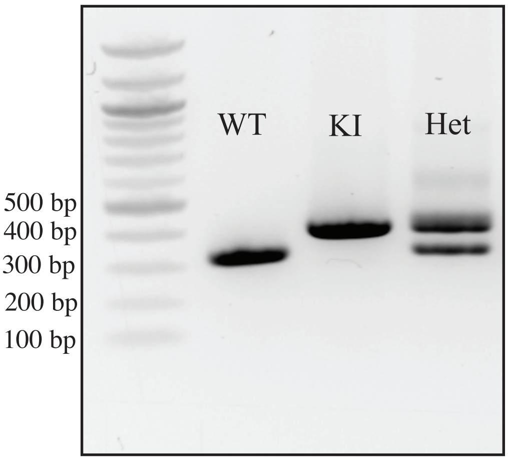

Supplementary Figure 2

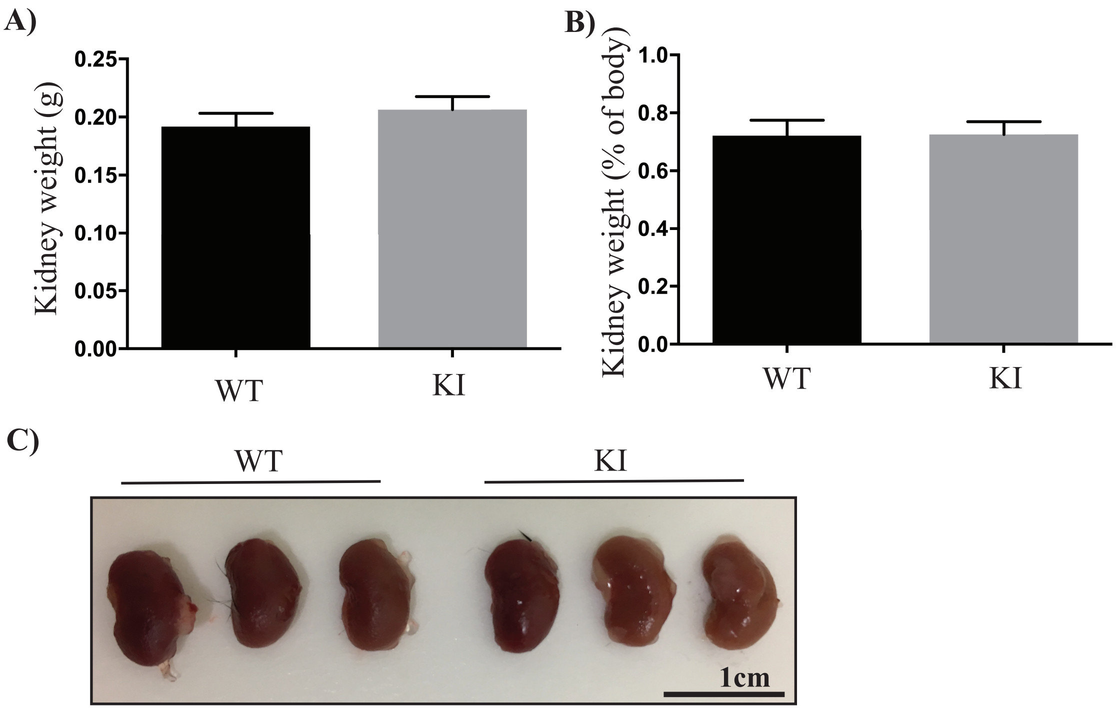

Supplementary Figure 3

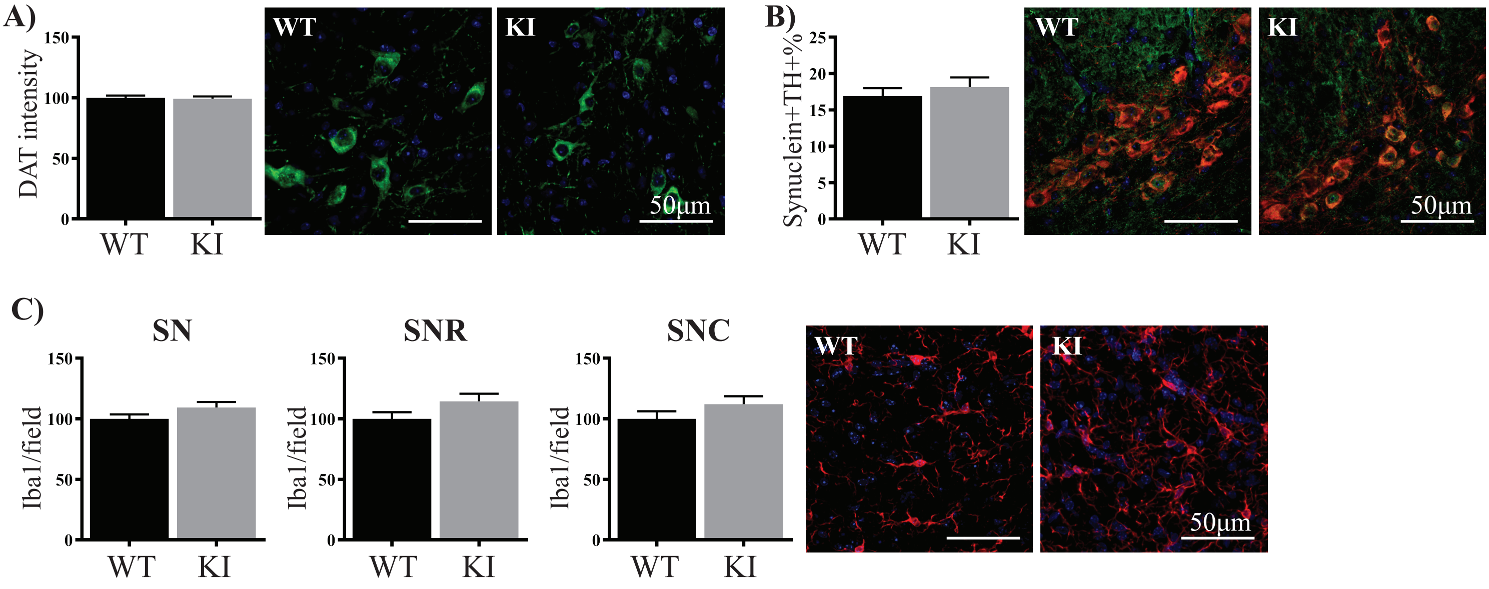

Supplementary Figure 4

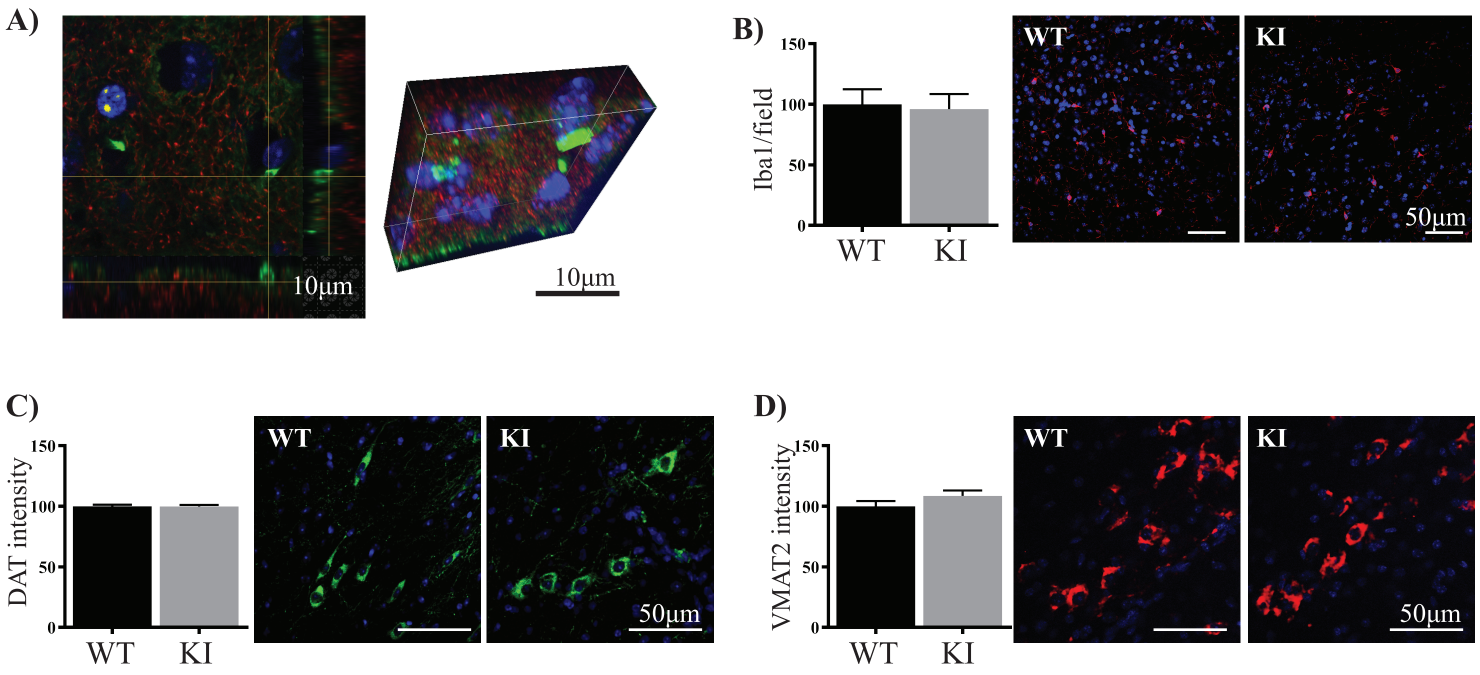

Supplementary Figure 5

A)

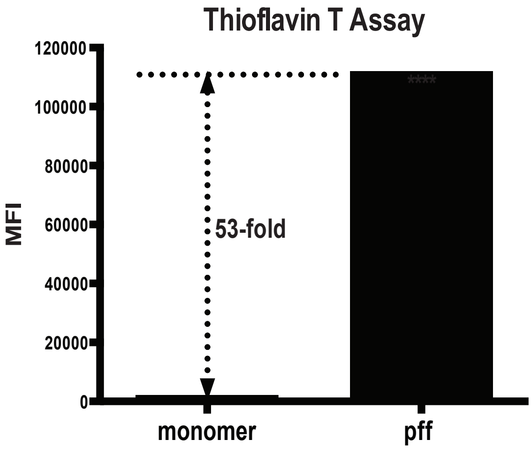

B)

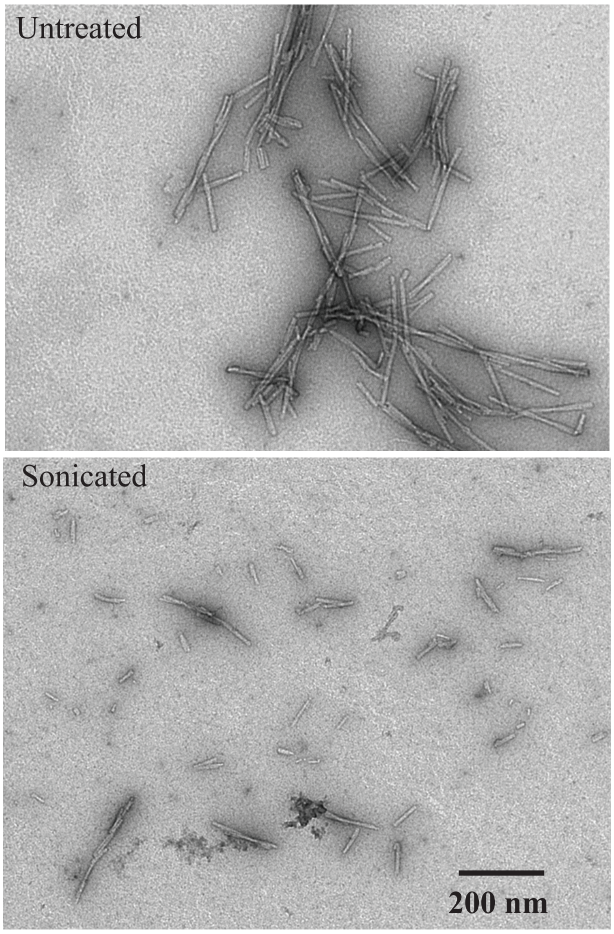

Supplementary Figure 6

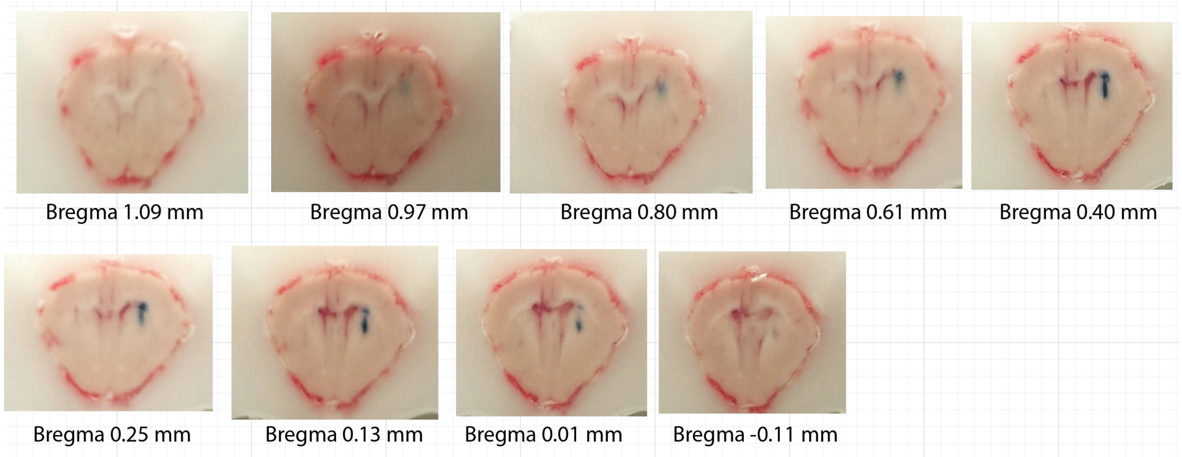

Supplementary Figure 7

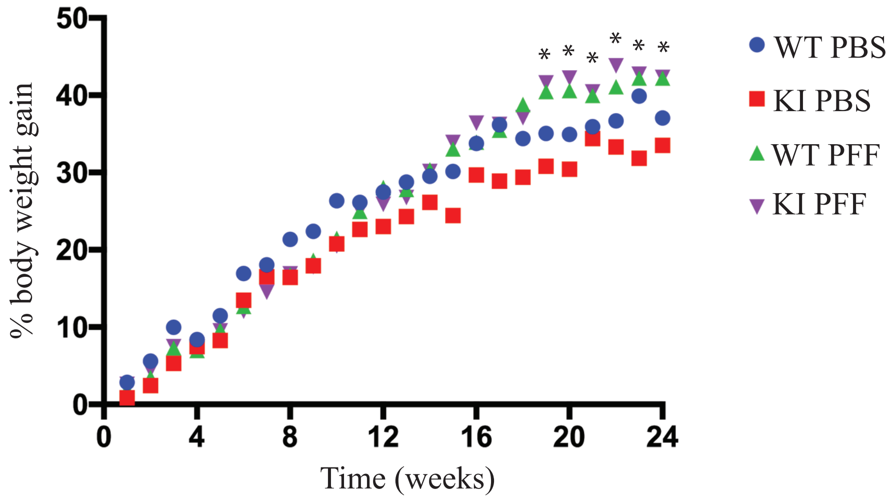

Supplementary Figure 8

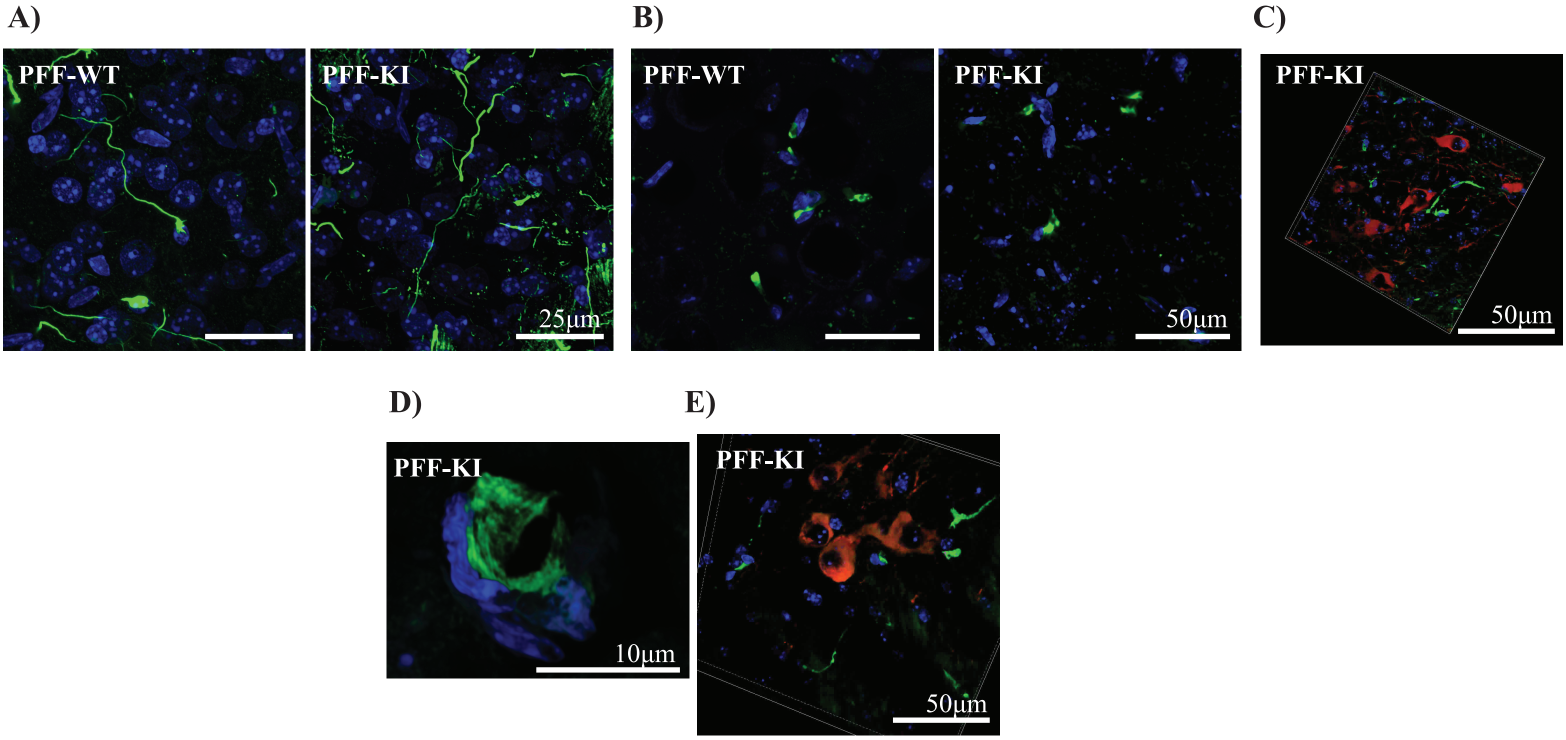

Supplementary Figure 9

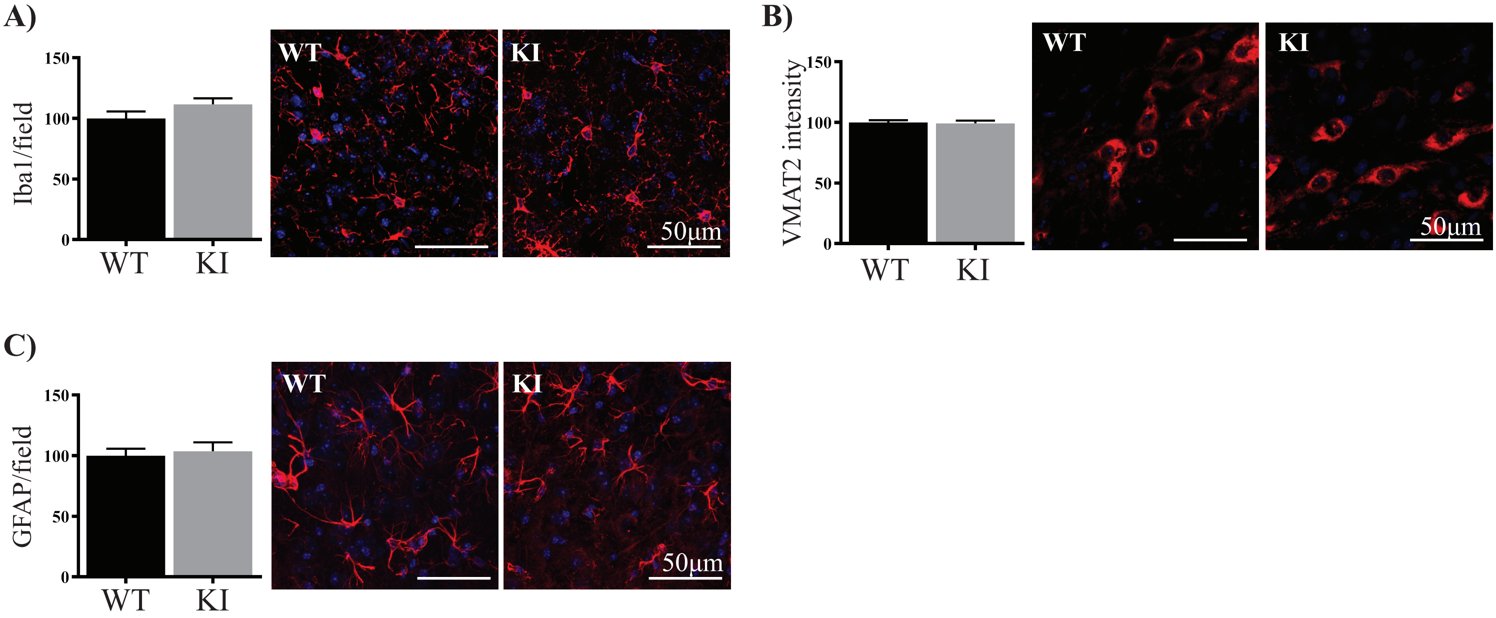

Supplementary Figure 10
